# Artificial Intelligence in Cancer Research: Trends, Challenges and Future Directions

**DOI:** 10.3390/life12121991

**Published:** 2022-11-28

**Authors:** Anu Maria Sebastian, David Peter

**Affiliations:** Department of Computer Science, Cochin University of Science and Technology, Kochi 682022, Kerala, India

**Keywords:** artificial intelligence in medicine, oncology, cancer diagnosis, cancer prediction, cancer treatment, cancer research, ethics, healthcare, machine learning, precision medicine

## Abstract

The World Health Organization (WHO), in their 2022 report, identified cancer as one of the leading causes of death, accounting for about 16% of deaths worldwide. The Cancer-Moonshot community aims to reduce the cancer death rate by half in the next 25 years and wants to improve the lives of cancer-affected people. Cancer mortality can be reduced if detected early and treated appropriately. Cancers like breast cancer and cervical cancer have high cure probabilities when treated early in accordance with best practices. Integration of artificial intelligence (AI) into cancer research is currently addressing many of the challenges where medical experts fail to bring cancer to control and cure, and the outcomes are quite encouraging. AI offers many tools and platforms to facilitate more understanding and tackling of this life-threatening disease. AI-based systems can help pathologists in diagnosing cancer more accurately and consistently, reducing the case error rates. Predictive-AI models can estimate the likelihood for a person to get cancer by identifying the risk factors. Big data, together with AI, can enable medical experts to develop customized treatments for cancer patients. The side effects from this kind of customized therapy will be less severe in comparison with the generalized therapies. However, many of these AI tools will remain ineffective in fighting against cancer and saving the lives of millions of patients unless they are accessible and understandable to biologists, oncologists, and other medical cancer researchers. This paper presents the trends, challenges, and future directions of AI in cancer research. We hope that this paper will be of help to both medical experts and technical experts in getting a better understanding of the challenges and research opportunities in cancer diagnosis and treatment.

## 1. Introduction

The term “cancer” was first used in medicine in the 1600s, which refers to cells that are developing abnormally and have the potential to infiltrate or spread to other parts of the body. Cancer is a complex and multifaceted disorder with thousands of genetic and epigenetic variations, especially in how they grow and divide. In a typical cell cycle, the cells go through the process of mitosis to reproduce themselves, leading to the cell’s normal growth. Eventually, the programmed cell-death process, known as apoptosis, causes the cells to die in order to ensure controlled growth. Once this process is disordered, the cells lose its balance and grow uncontrollably to form malignant tumours invading the surrounding tissues. The cancer cell may potentially move through the bloodstream or lymphatic system to different organs of the body and continue to spread from there. There are two types of cancer cells, namely benign and malignant. Benign cells do not spread to other parts, while malignant cells metastasize and are considered to be more destructive. There are more than 500 genes known to be connected to different forms of cancer. Cancer is one of the major causes of deaths in the world, especially for adults under 70 [1]. One of the world’s most rapidly developing therapeutic fields nowadays is oncology, and there is a need to accurately diagnose cancer at an early stage to enhance patient’s survival rate. The current research efforts are focused on cancer etiology and therapy, resulted in huge repositories of cancer-associated data which could be used for cancer prediction and early detection [2]. The lengthy and expensive treatment methods for cancer are due to the disease’s high mortality and recurrence rate. Because of the developments in translational research and clinical trials, cancer has the highest number of clinically relevant mutations and the greatest multimodality-therapeutic options. Given the wide variety of cancers and the variety of observed manifestations, oncology may have the highest demand for customised care.

AI intends to mimic human-like cognitive abilities to address difficult healthcare challenges, including complex biological abnormalities like cancer, through algorithms and programs with appropriate data. These algorithms are designed to have a set of instructions which analyse data to take an appropriate decision or course of action [3]. Since the human mind is only able to analyse a finite amount of data in a short amount of time, the exponential rise of AI over the past ten years is a proof that it can serve as a platform for supporting human experts to make the best possible decisions. AI-based algorithms hold great promise to pave the way to identify genetic mutations and aberrant protein interactions that can lead to cancer at a very early stage. One of the goals of current biomedical research is to ethically and safely introduce AI technology into medical settings. The greatest advancement in disease risk, diagnosis, prognosis, and therapy prediction may come from AI-based support for pathologists and doctors. The future of medical guidance will move toward speedier mapping of a new treatment for each individual through clinical applications of AI and machine learning (ML) in cancer diagnosis and treatment. Researchers may work together in real-time and share information digitally with AI to possibly treat millions of people. In this paper, healthcare and AI are combined in an effort to showcase clinic technology that is changing the way people think about healthcare and to show how AI-based support enables medical practitioners to deliver accurate care. The requirement for this expertise is crucial since a technology that a medical professional is unfamiliar with has no bearing on healthcare [4].

Some major problem classes for AI-interpretation tasks include speech recognition, time-series analysis, computer vision, and natural language processing (NLP). For instance, radiographic-image interpretation can benefit from computer vision. Time-series analysis, on the other hand, is helpful for analysing continuously streaming health data from an electrocardiogram. Similarly, AI-based NLP can be useful in the extraction of pertinent information from electronic health record (EHR) data, and speech-recognition algorithms can be employed for the identification of neurological problems [5].

## 2. Cancer Statistics

The International Agency for Research on Cancer (IARC) estimates that globally there are 36 types of cancer. They discovered that one in five people will develop cancer at some point in their lives, and one in eight men and one in eleven women will pass away from it. Around eight million people die each year of cancer [6,7,8]. According to GLOBOCAN 2020 statistics, the cancer with most incidence rate in women is breast cancer and in men is lung cancer [9]. According to Center for Disease Control and Prevention (CDC), among the types of cancer, the highest deaths were caused due to lung cancer, accounting for 23% of all cancer deaths. The WHO reports in 2022 [6] that cancer accounted for about 10 million deaths worldwide in 2020. Table 1 shows the worldwide cancer-related deaths in 2020. Table 2 depicts the most common new cancer cases worldwide in 2020. WHO reports that about 400 thousand children develop cancer worldwide every year.

## 3. Conventional Practices for Cancer Diagnosis and Treatment

Traditionally, a patient approaches a healthcare provider when they have a health issue that is accompanied by symptoms like hard lumps on the body or odd patterns on the skin. As the first step in the cancer-detection procedure, the clinic gathers the patient’s clinical history, screening tests, and medical imaging. The screening test seeks to identify individuals with a specific cancer or pre-cancer who have not yet shown any symptom and, if found, swiftly direct them for additional testing and treatment. A pre-stage analysis may be performed using a variety of scan modalities. This is done as a precaution to prevent cancer in a high-risk population from being diagnosed too late. Following a suspicious finding, the tissue samples from the concerned area are taken and evaluated in a lab. Medical professionals are consulted on the findings for further insights [10,11]. They gather, combine, and interpret the related information while also making a diagnosis recommendation. The patient is informed of the working diagnosis and prognosis, and the proper course of therapy is prescribed. This process is beneficial for both the patients and the healthcare system, with facilities for quick diagnosis and abilities to learn from mistakes. However, this process leaves potential for committing errors while also being flexible depending on the medical specialty.

A qualified clinician uses a variety of medical images to visually inspect potential areas that mirror the indications of malignant tumours in order to make a non-invasive diagnosis of cancer. The clinician’s experience has a significant impact on how accurate the diagnosis is. Since reading these scans accurately requires years of training, and radiologists often differ when examining the same scan. Furthermore, this approach is tedious and not consistent due to the sheer size of the medical information. The time that medical specialists can commit to making a diagnosis is typically constrained, and it may be difficult to draw conclusions from non-standardized data from a variety of modalities. Additionally, as a diagnosis requires input from a number of specialists across a range of medical specialties, the process may take longer than expected [12]. Conventional cancer treatment is challenging as it requires patient-specific therapy combinations to be tried and tested. The main methods used to treat individuals with malignant diseases include mechanical, physical, chemical, and biological therapies.

One or more traditional modalities, such as chemotherapy, surgery, and radiation, are included in the prescribed-customised treatment plan. Chemotherapies, a type of systemic therapy that is given through the bloodstream, can be used to treat malignant diseases at any anatomic site in the body. When medicine needs to be given systemically throughout the body, it is the only option for treating metastatic sickness. Chemotherapy uses drugs that target rapidly dividing cells as its primary means of killing malignant cells. There are harmful side effects from the medications used to reduce tumours. A few specific hormones’ effects on the body are the basis of hormone-level therapy [13,14]. When it comes to patients with prostate or breast cancer, hormones play a significant role. Immunotherapy tries to boost the immune system’s ability to combat malignant cells. Examples of immunotherapy include checkpoint inhibitors and adoptive-cell transplants. By altering their deoxyribose nucleic acid (DNA), radiation therapy either eliminates malignant cells or inhibits their growth. This therapy is frequently suggested by medical professionals as a preoperative measure to reduce cancer symptoms or shrink tumours. Photodynamic treatment, radiation therapy, hyperthermia, and heat therapy are all physical methods of tumour eradication. Blood-related cancers like leukaemia and lymphoma can benefit from stem-cell transplantation. The procedure entails the removal of blood cells that chemotherapy has damaged. Utilizing targeted therapy helps boost immunity and prevent the spread of cancer. Examples of target therapy include monoclonal antibodies and small-molecule medications.

Surgery is a mechanical method of removing tumours. Surgery is the most popular choice, because it provides the greatest possibility for success. It is a medical strategy that entails tissue removal, analysis, and repair. Only solid tumours that are contained to a single area respond well to this local therapy. Surgery is typically performed on patients who have malignant cells in their bodies. In addition, the disease’s spread can be controlled by removing the lymph nodes.

It might not always completely eradicate every malignant cell within the patient, and therefore, the tumour might return over time. The current methods of cancer detection and treatment have disadvantages, such as higher percentages of false-positive test findings that imply someone has lung cancer when they actually do not. For instance, some noncancerous lung alterations appear very much like cancer on CT scans. Also, an early diagnosis can cause, for instance, some of the cancerous nodules in the scan to not be visible to the naked eye. Increased combination of treatment trials and associated expenses increase the pain of treatment suffered by the patient. The risk of repeatedly exposing patients to radiation for routine screening is real [15]. Advances in AI has proved to minimize the drawbacks in conventional cancer diagnosis and treatment, and has the potential to bring revolutionary changes in cancer healthcare.

## 4. AI for Cancer Research

Since the field’s inception, experts have predicted the potential of highly tailored oncology care employing AI technologies. This promise is being realised as a result of cumulative advancements in the sciences, including the improvement of ML and deep-learning (DL) algorithms, the expansion of the breadth and variety of databases, including multiomics, and the decline in the price of massively parallelized computing power.

Fuzzy logic and neural networks are the two main methods used by AI to mimic human intelligence. In contrast to fuzzy-logic models, the results of neural-network models are very difficult to interpret and are referred to as “blackbox” models. While the data-driven AI (DAI) paradigm is guided by data, the symbolic AI (SAI) paradigm is guided by human-domain expertise. Most often employed in deterministic situations, SAI joins human-readable symbols in a relationship akin to “if-then” expressions to draw conclusions. In order to help solve situations where simple rules are sufficient, SAI explicitly incorporates human knowledge and rules into computer systems. This allows computers to reason and arrive at educated judgments. In other words, while DAI uses historical data as experience to develop mathematical equations that generate intelligent decisions, SAI focuses on reasoning with rules specified by human experts, requiring little to no learning. SAI and DDAI concepts are combined in informed AI (IAI). In order to create the target variable (i.e., data annotation) and make the models explicable, SAI takes into consideration human-domain expertise. DAI has a significant function to play in the study of cancer [16].

It requires time and special technologies to ensure data security and privacy while making inferences from encrypted data. The Stained-Glass Transforms used by Protopia AI reduce the risk of sensitive data leakage when drawing conclusions from data, which is frequently a barrier to using the data for ML and AI. These transformations work well with many different sorts of data, including tabular, text, picture, and video. Prior to therapeutic treatments, it may anticipate prognostic markers, including patient outcome, pharmacological efficacy, and resistance, providing a very credible foundation for following therapies and providing a customised scenario [17].

Researchers have developed tools to help with cancer identification and prognosis as a result of the availability of open-source healthcare statistics. To address problems in cancer medical treatment, DL and ML models offer dependable, quick, and efficient solutions on distributed dataset. For the analysis of distributed data, advanced-federated learning models can be deployed. Whole-blood, multi-cancer detection using deep sequencing, virtual biopsies, and NLP to infer health trajectories from medical records, and advanced clinical-decision support systems that incorporate genomics and clinomics, are some of the emerging clinically useful techniques [18]. Oncology heavily relies on evidence-based, medicine-scoring systems for cancer-risk assessment, disease diagnosis, prognostic staging, treatment, and surveillance monitoring. These systems began as straightforward light-microscopy observations and improved to more sophisticated testing, such as gene-expression tests and next-generation sequencing of somatic and germline genomes. AI also opened doors to the synergic usage of drugs for cancer treatment.

**Figure 1:**
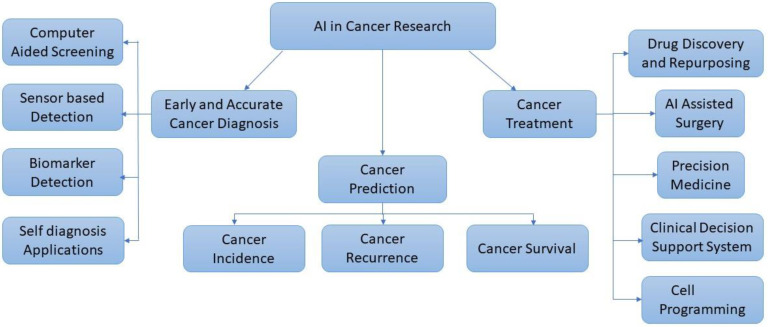
Approaches for cancer research using AI.

When backed by strong AI core services and resources, AI-powered cancer research is approachable even for those without much computer knowledge. The future of digital healthcare and clinical practices is anticipated to shift toward the usage of algorithm-based AI for radiological image interpretation, EHRs, and data mining to give a more precise cancer therapy. Marketing-cancer-research companies have estimated the cost-savings from intelligent AI applications in the US healthcare sector to reach $52 billion in 2021 [8]. The AI intervention in cancer research can be more effective if proper data are available for developing the ML and DL models. Figure 1 shows the broad approaches for cancer research using AI.

### 4.1. Cancer Data Repositories

Software, data, technology, and services are all part of digital health, which applies digital transformation to the healthcare industry. According to Deloitte Insights, radically interoperable data and AI promise consumer-focused and prevention-oriented healthcare. Availability of data is essential for data-driven AI research and many researchers develop frustration due to the lack of adequate data to carry out their research [19,20].

To uncover ways to enhance cancer treatment, care, and prevention, cancer experts are continually developing new clinical trials. To assist people in locating a research study that might be suitable for them, many organisations provide online lists of open clinical trials. The following tools, lists, and searchable databases are useful for locating a cancer clinical [21].

- Ancora.ai.
- Be the Match: Jason Carter Clinical Trial Search and Support Program
- Bladder Cancer Advocacy Network
- BreastCancerTrials.org
- Center for Information and Study on Clinical Research Participation (CISCRP)
- CenterWatch
- ClinicalTrials.gov.
- EmergingMed Clinical Trial Navigator Service
- Lazarex Cancer Foundation
- Melanoma Research Alliance
- Metastatic Breast Cancer Project
- Metastatic Prostate Cancer Project
- National Brain Tumor Society Clinical Trial Finder
- National Cancer Institute (NCI) Clinical Trials
- Pancreatic Cancer Action Network Clinical Trial Finder
- SPOHNC Clinical Trial Navigation Service
- Targeted Agent and Profiling Utilization Registry (TAPUR) Study
- The Leukemia & Lymphoma Society Clinical Trial Support Center
- Us TOO Prostate Cancer Clinical Trial Finder
- World Health Organization (WHO) International Clinical Trials Registry Platform.

#### 4.1.1. Types of Cancer Data Repositories

For computational analysis of cancer, a variety of data sources are available, including electronic health records (EHRs), diagnostic imaging, pathology slides, and peripheral blood counts. Here we present a quick overview of the main forms of data utilised in cancer research to generate new insights.

#### 4.1.2. Radiographic Images

- Cancer Imaging Program (CIP) [22]: The National Cancer Institute’s (NCI) CIP supports and promotes basic, translational, and clinical imaging research related to cancer, as well as the integration and application of these imaging advancements to the study of cancer biology and the treatment of cancer and cancer risk.
- Cancercentre.ai [23]: It contains data like screenshots from the radiology platform that depict MRI of the prostate (T2-weighted images in axial plane). It provides trained radiologists with pre-screened images and identified features.
- The Cancer Imaging Archive (TCIA) [24]: TCIA has a sizable collection of medical photographs of cancer that are available for download by the general public. The image modality, or image type supplied are MRI, CT, digital histopathology, etc.

Oncologic detection, diagnosis, therapy response, and prognosis have all benefited from the use of radiomic characteristics, which can assess tumour intensity, shape, and heterogeneity. Computer-aided detection (CADe) or computer-aided diagnosis (CADx) in clinical radiography imaging is the system-based framework that supports specialists in making choices quickly. MRI-derived Digital Imaging and Communications in Medicine (DICOM) pictures can aid in the diagnosis and segmentation of cancer with higher prediction accuracy. A Computed Tomography (CT) scan was utilised to train the model in about 23% of the literature. Additionally, a lot of research has used pathological, endoscopic, and mammographic imaging. The classification is made more challenging by the low contrast of CT-scan images, which makes it difficult to distinguish the object from the backdrop. A CT scan hardly ever picks up certain tumours, like prostate cancer and certain liver cancers [3,25,26].

Lung, breast, brain, and prostate cancers are among the tumours for which radiographic evaluation is frequently used. It largely relies on visual assessments, while advanced computational analysis may be used to enhance its interpretations. AI, in particular, promises to make significant advancements in the qualitative interpretation of cancer imaging by expert clinicians, including volumetric delineation of tumours over time, extrapolation of the tumour genotype and biological course from its radiographic phenotype, prediction of clinical outcome, and assessment of the impact of illness and treatment on nearby organs. The clinical workflow of radiographic identification, management choices regarding whether or not to provide an intervention, and ongoing observations may change to a yet-to-be-envisioned paradigm as a result of AI-automating activities in the first interpretation of radiographs. In task-specific applications, DL has been demonstrated to match and even outperform human performance by automatically learning feature representations from sample images. Additionally, DL techniques offer improved robustness to noise, higher generalizability across diseases and imaging modalities, and a reduction in errors that will ultimately result in earlier treatments and major advancements in clinical care and diagnosis [27,28,29]. Although the majority of these studies still fall under the preclinical-research category, the continuous development of these automatic-radiography indicators may reveal clinically relevant alterations in tumours and lead to a paradigm shift in the long-term classification of cancer.

Object localisation in radiography is referred to as detection. To minimise observational errors of omission and act as a first line of defence against them, DL-based detection methods can be deployed. It can spot indeterminant nodules, and in a broad sense, characterization includes tumour diagnosis, tumour staging, and the degree and segmentation of abnormalities [30,31,32]. Clinically, such issues are resolved using subjective, qualitative qualities, but CADx systems use a systematic analysis of quantitative-tumour data, allowing for more repeatable descriptions. Strong tumour descriptors are provided to capture intra-tumor heterogeneity. Although many of the problems with lung-cancer screening have been resolved using traditional biostatistical and machine-learning methods. Such methods may be replaced by AI in order to find biomarkers that better distinguish between benign and malignant nodules, minimise imaging false-positive results, and reduce false-positive imaging results [27,33,34].

The pipeline for detecting cancer using radiography includes the steps of detection, localization, segmentation, registration, and classification. By providing radiologists with more accurate and consistent measures, AI researchers are able to increase their level of knowledge. This is done through computer vision and ML. Analysis of 3D scans pixel- by-pixel provides the radiologist with precise information regarding the tumor’s growth, shrinkage, or shape change from the previous scan. In the literature, convolutional neural network (CNN) (41%), conventional neural network (10%), support vector machine (SVM) (9%), deep neural network (DNN) (8%), and ensemble models (8%) make up roughly 76% of the models employed for cancer detection from radiographic images [3].

Since training the model on large-sized photos is frequently computationally and time-inefficient, the images are first converted after being resized. To assess the model’s performance, the data are then divided into training and validation sets. Three convolutional layers, each followed by a maxpooling layer, can be used to construct a basic-sequential-CNN model. It takes the low-level, mid-level, and high-level traits, and extracts them. In the pooling procedure, each channel of the feature map is covered by a two-dimensional filter, and the features that are present in the filter’s coverage zone are summarised. After that, a flatten layer is added to flatten the output of the convolutional layer. Two fully linked layers are added after that, followed by BatchNormalization layers to facilitate quick and reliable training, and a Dropout layer before the final layer is added to prevent the potential of overfitting. The final layer is what provides the categorization with the final soft probability. The three key parameters that must be carefully designed while building a model are the optimizer, loss, and metrics. To determine whether the model is getting better with each epoch, callbacks can be employed [27]. Successive layers of a computer-vision algorithm pick up on a variety of information, such as the ability to recognise edges in an image, patterns of edges that indicate forms, groups of shapes that indicate certain items. Medical-imaging- abnormality identification, differential diagnosis, and worklist prioritising are all made possible by AI-enabled diagnostic imaging interpretation.

#### 4.1.3. Genomic and Molecular Data

- The Cancer Genome Atlas (TCGA): Over 20,000 cancer samples from 33 different cancer types are characterised as TCGA, a popularly utilised public database for cancer research that produces numerous types of data and tools [20].
- International Cancer Genome Consortium (ICGC) [35]: In 50 of the most significant cancer forms, it maps the genetic flaws. Worldwide cancer researchers are given free access to all the data from the 25,000 cancer samples that were examined.
- cBioPortalData [36]: It allows visualization, analysis, and import of cBioPortal datasets as MultiAssayExperiment objects in Bioconductor.
- Genomics of Drug Sensitivity in Cancer [37]: It characterizes over a thousand human cancer cell lines, and hundreds of chemicals are tested on them. It also offers information on drug response as well as genomic-sensitivity markers.
- Cancer Cell Line Encyclopedia (CCLE) [38]: This is useful for investigating cancer biology, identifying cancer targets, and determining treatment efficacy.
- LinkedOmics [39]: It offers a platform for accessing, analysing, and comparing cancer multi-omics data inside and across tumour types.

The numerous obstacles keeping tailored treatments at arm’s length can cast a shadow over precision medicine. The ability to gather, examine, and make use of patients’ genetic data is the main obstacle. AI is being used more and more by providers and researchers to glean useful insights from genetic data. The first stage in transforming genomic data from an unintelligible resource to a useful medical asset and one that will heavily rely on AI is turning precision-medicine treatments into a reality. AI is able to comprehend the diverse pathology of cancer with comparable symptoms [5]. The underrepresentation of some groups in the statistics, however, can result in a conclusion that is not in the best interest of all patients.

By identifying patterns throughout the entire transcriptome, ML/DL algorithms can get around the limits of conventional computational techniques. The feature selection approach is a crucial step in deciding the AI performance while processing complicated variables. The huge data dimension makes processing more challenging, particularly for high-throughput omics. The most important variables can be found after feature selection, which reduces dimensionality, cuts training time, improves the model’s capacity to generalise, and prevents overfitting. AI models are guaranteed to perform better when they use a reliable feature-extraction method. Numerous clinical-genomics tasks, including variant calling and annotation, variant-impact prediction, phenotype-to-genotype mapping, can be completed using AI [5].

Genomic data have several levels, from the raw data produced by the instrument at first to processed and normalised data ready for more in-depth studies. Level 1 (raw data) to Level 5 are the possible levels of molecular-profiling data. The degree of molecular profiling determines the degree of automation for analyses. Level 5 requires additional human involvement and innovative analysis to generate insights. A high level of knowledge in bioinformatics analysis is required for delving further into genetic databases. Collaborations between molecular biologists who can offer fresh experimental-model datasets and domain knowledge are highly feasible [40]. Genome wide association studies (GWAS) have effectively found genetic variations interacting to increase the risk of cancer [41]. Models based on single data types risk missing significant predictive information originating from the interaction of interdependent biological systems due to the complexity of cancer biology. A concentrated effort can be made to investigate life using new generation sequencing (NGS), multiomics, which include the genome, proteome, transcriptome, epigenome, metabolome, and microbiome, depending on how they are sequenced.

The relationship between certain problem classes and diagnostic tasks by AI may not always be obvious. For instance, computer-vision techniques are helpful for the identification of functional-regulatory elements in the human genome, where it spots the recurrent motifs in DNA sequences in a way similar to how CNN detects pixel patterns in images [5]. Many of these issues have been solved by DL, which use intricate network structures to extract interpretable features from massively complicated datasets. The input-DNA sequence is divided into subsamples by CNNs, which then apply masks to the data from the subsamples and multiply each feature value by a set of weights. The results show patterns that relate to the initial sequence. These feature maps can be used to train a classifier to predict a given label using a feedforward- neural network or logistic regression. By masking some base pairs and retaining others in each permutation, it is possible to identify motifs that are more crucial for correctly classifying the sequence. A segmented-DNA sequence is provided to recurrent neural networks (RNNs), which use connected hidden states to find connections between input units. Unidirectional-hidden-recurrent nodes that only convey information in the forward direction while reading the input sequence are frequently used to represent the hidden states. Another option is to employ a bidirectional RNN, which scans the input sequence and transmits concealed state data both forward and backward. Each input unit’s context is inferred based on its hidden state, which is influenced by the hidden states of its nearby input units, as well as the anticipated context labels for those units, such as position versus direction, or intron against exon [5,42,43].

Extensions of this idea, used in specialised networks like long short-term memory networks (LSTMs), add network components that improve the network’s capacity to memorize long-term dependencies in the input data. The main benefit of these AI algorithms is their enhanced capacity to identify non-linear or multi-step interactions that are not effectively probed by conventional methods like hidden Markov models [44,45]. AI-time-series algorithms seem to be particularly good at identifying functional-DNA-sequence elements that are suggestive of large-scale regulatory elements, gene function, and gene splicing when applied to genomic-sequence data. Targeted medicines may be hampered by current difficulties, despite the fact that the complexity of genomic data makes this a perfect application for advanced analytics techniques. When utilising AI in cancer to evaluate genomic data, there is still a risk of bias, inaccuracy, and a lack of education and training [46]. In order for the research to achieve the objective of personalization in medicine, these issues will need to be resolved.

#### 4.1.4. Pathological Images

- Whole Slide Imaging Repository [47]: Pathology departments use scanned photos of traditional glass slides to create digital slides as their imaging method.
- Cancer Digital Slide Archive [48]: It hosts pathological images maintained by TCGA.

The science of pathology involves identifying diseases mostly by analysing tissue, cell, and bodily fluid samples. Pathology has a number of diverse subspecialties that pertain to various tissue types, such as the prostate, lung, and breast. The diagnosis of cancer or a benign condition is typically made using Hematoxylin and Eosin (H&E) slide images [49]. Systems for pathology AI must be created to give tissue extensive, general-purpose information that may be used as the basis for a variety of specialised interpretations or scoring methods. Any pathology-AI system must complete the crucial task of cell classification.

The use of AI- and ML-based computational tools is increasingly important in digital pathology. It can make use of the DNN models to create precise tumour pictures with high resolution and new biomarkers. Biomarkers are the biological signatures that can identify the tissues and cells of the human body. The conversion of histopathology slides into high-resolution images by slide scanners is the basis for the digitalization of medical pathology, which is a significant breakthrough for enhanced cancer diagnoses [4]. Digital whole slide images (WSIs) can ensure the comprehension of biological complexities in cellular architecture and are currently being processed using ML systems for DL analytical processing. A fully automated AI is not advised due to the variations in pathology between different patient types. Treatment decisions, which include the complete spectrum of possible drugs and the rich information data for tissues, would allow better characterization of patient populations, leading to smarter tissue- and patient-selection strategies that would accelerate and lower the costs of new drug developments. Any pathology AI system must contend with variances among various patient kinds. No two patient samples have the same appearance during a disease state. It can be observed that the same cell type exhibits various, frequently incongruent, characteristics in different patients, which is something that any ML system must handle in order to distinguish between different cell types [49].

Despite extensive clinical and pre-clinical testing, many biomarker-based medicine developments today face a number of challenges, including significant failure rates. There is a critical need to find a new generation of biomarkers using computation techniques for major clinical practice and medication discovery in order to increase the success rate.

#### 4.1.5. Clinical Data and Blood Profiling

- Data-CAN [50]: It is a cancer-data-knowledge network that was co-created in order to produce better results and greater social benefits for cancer research.
- Optum Oncology EHR data [51]: Optum data include vital signs, symptoms, problem descriptions, clinical evaluations, lab results, techniques, diagnoses, surgeries, and therapies. Additionally, it contains information about the cancer’s stage, grade, histology, genetic mutations, other blood biomarkers, lines of therapy, and assessments of the disease’s development and drug response to further increase its usefulness to oncology.

Clinical data include information on demographics, clinical traits, investigations, specifics of the treatment, its results, and other outputs. It is critical to distinguish between national-public-health-data registries and local-hospital-EHR data. EHR data often consists of free-text, unstructured clinical notes and reports, as well as structured, easily quantifiable information like admission dates or blood results. Utilizing NLP techniques, the latter can be analysed. Up to 80% of medical records are unstructured, according to big data analytics in healthcare, and as a result, they are mostly underutilised because mining and extracting these data are difficult and time-consuming. Data cannot be extracted by current computer-based methods without NLP technology [52]. For uniformity among institutions, registers are used to establish unified database architecture. Long-term evaluation of patient growth, development, and recovery are made possible by advancements in medical-record databases, such as upgrading of EHR systems. Moreover, the expansion of access to powerful computing resources is mandatory for the developments in AI. These improvements, along with numerous others, have opened up fresh study directions and chances for interdisciplinary cooperation. NLP converts unstructured free-text into a machine-analysable format, enabling the automation of labor-intensive processes. Studies have demonstrated that blood profiling, which involves AI algorithms analysing plasma circulating tumor DNA (ctDNA) and micro-ribonucleic acid (miRNA) profiles, is a more effective way to detect and monitor cancer than the conventional CT scans [53]. A cutting-edge AI-based technique was created by researchers at the Johns Hopkins Kimmel Cancer Center to detect lung cancer using blood testing.

Using EHRs as their input, NLP algorithms modify the documents in a productive sense. The capacity for computer-assisted coding to condense the content of lengthy chart notes into only the key elements is a clear benefit of implementing NLP in EHR. Language translation, document categorization, document summarising, or the extraction of the text’s higher-level concepts are only a few examples of this transformation. To extract semantics or identify named entities from text, typical NLP algorithms first analyse the text’s syntactic structure. Some of the well-known NLP models for text processing are RNN and LSTM. The range of expressions that can be employed to describe a single idea, including phrases, synonyms, and related concepts, is a significant challenge that NLP attempts to address [52,54]. Because regulated vocabularies are vast and constantly changing in clinical applications, this issue is particularly severe.

### 4.2. Using Cloud AI Platforms

The growth of zero-code AI platforms make it possible for health specialists to use AI without having any programming experience by using APIs. The establishment of cancer-related AI relies on the trifecta of computational algorithms, databases, and computing power in an effort to make use of the inherent data richness of the subject. To achieve the level of precision that oncology strives for, each of these AI principles must be expanded beyond its existing limitations. Medical professionals across the world are exchanging information about disease diagnosis and management, drug response, and strategies for managing diseases with precision. Moreover, such data might be automatically stored to cloud and utilize its computing power for discovering insights with AI. Cloud computing is essentially the use of remote-internet-connected servers which can be used to store and process data. Google, Amazon and Microsoft are the major cloud-AI-platform providers. The AI-cloud platform allows the training of ML and DL models with a wide range of customization options. Cloud is very convenient for healthcare institutions to store massive amounts of data securely, analyse them and get useful insights. This has led to the establishment of Tumor Atlas [20,55].

Microsoft’s significant investment in cloud computing makes sense for a subject that requires a lot of computer power to tackle challenging issues. Jasmin Fisher, a biologist from Microsoft Cambridge, U.K., developed a Bio Model Analyzer (BMA), a cloud-based application [56] that makes it possible for biologists to simulate how cells interact and communicate with one another as well as discover the connections they form. Literome is a cloud-based method for organising genetic research that could be useful for cancer diagnosis. Due to the volume of data, oncologists would find it challenging to complete their duty alone. Hoifung Poon, a researcher at Microsoft’s Redmond, Washington lab used ML to generate sophisticated models for locating various descriptions of identical knowledge in order to produce Literome, which uses NLP capabilities that only need a modest quantity of knowledge to be available [56].

BigQuery, a highly scalable, multi-cloud data warehouse from Google Cloud, supports the cloud-based platform that connects researchers to a large number of cancer datasets and provides the analytical and computational infrastructure to quickly evaluate that data. Medical researchers can now directly study data on the Institute of Science Biology-Cancer Genomic Cloud (ISB-CGC) platform without having to download it using Google Cloud services like Notebooks and BigQuery application programming interfaces (APIs) [57]. The success of ISB-CGC in leveraging Google Cloud as the cornerstone of their infrastructure and data-cloud strategy has allowed the cancer-research community to receive secure, real-time access to data that is crucial to the early diagnosis of cancer.

### 4.3. AI for Cancer Prediction

Predictive models powered by AI are now a crucial component of cancer treatment. Predictive models can establish a person’s likelihood of getting a certain cancer by identifying the risk factors. Before the disease takes hold, AI can recognise those who are at high risk of contracting it. This enables medical professionals to keep a close eye on these patients and act quickly when necessary. DL, according to researchers at the University of Hawaii, can tell apart between mammograms from women who would later get breast cancer and those who will not. The Massachusetts Institute of Technology (MIT) researchers have developed a DL model to predict cancer risk from mammogram images. They validated the model using data from several hospitals across different continents. The algorithm correctly identified 30% of future breast cancer patients as belonging to a high-risk group. On the other hand, human doctors who used the traditional Tyrer-Cuzick methodology only flagged 18% of the cases [4,53].

Different research teams have created random-forest-ML models to predict cancer survival and long-term cognitive outcomes [58]. Identifying the biological mechanisms necessary for proper growth and how they affect the state of the learning tissue is crucial here. It is, therefore, exceedingly challenging for a human to measure the shift, but a machine may analyse millions of these photos from many modalities holistically to draw conclusions. In order to better personalise treatment regimens and provide better patient counselling, prediction of overall survival, recurrence risk, or other outcomes for cancer patients would be helpful.

### 4.4. AI for Cancer Diagnosis

The NYU Langone’s Perlmutter Cancer Center started using their AI classifier for cancer diagnosis in October 2019. This classifier can help pathologists in diagnosing cancer more accurately, reducing hospital error rates. The AI-based cancer classifiers, in general, can recognize patterns that are too subtle for the human eyes to detect. This will help physicians to perform targeted cancer therapies for patients with improved outcomes [55]. The classifiers are normally trained using thousands of cancer samples, which could be much more than a single pathologist can see in a lifetime. Moreover, the machines will be consistent and will not be affected by inter-reader variability. Thus, AI- based systems can be used even by inexperienced radiologists to get clear diagnoses, leading to the right treatment. Researchers from the Korea Institute of Science and Technology (KIST) have developed a neuromorphic technology for cancer diagnosis which combines AI, IoT, and autonomous technologies. They used tactile-neuron devices with artificial neural network (ANN) based learning methods. When pressure is applied to a potentially cancerous site, the generating electrical spikes will increase or decrease depending on the stiffness of the object or tumor encountered.

It is envisaged that ongoing research to assist the application of AI to cancer genomes would enable multicancer early detection and tumour-site-origin determination. This may improve cancer survivors’ surveillance plans and change cancer screening, particularly for less common and rare tumours [59]. Both scientists and physicians are becoming more and more interested in the field of radiology. The use of radiomics for diagnostics, prognostics, and treatment decision processes is becoming more and more appealing due to developments in pattern recognition, computer vision, and model building. As a potential non-invasive method of disease-heterogeneity quantification that could be used in conjunction with invasive biopsies and conventional quantitative-imaging techniques, radiomic features have the potential to quantify information about the entire tumour as well as the various textures contained within that tumour. These initiatives also make it easier for patients who are asymptomatic to get a cancer diagnosis. The creation of biosensors, which are non-invasive or implanted, may now identify the presence of cancer-related biomarkers. The three major parts of a biosensor are typically a biological sensing element, a physiochemical detector or transducer, and a signal processing system. Increased interest in the use of implanted medical devices for customised medicine has been brought about by developments in electronics and technology. The implanted biosensor can be used to monitor tumours as well [59].

### 4.5. AI for Cancer Treatment

Though surgery, chemotherapy, and radiotherapy will continue to be the gold standard for cancer treatment for a very long time, concerns like how quickly a tumour is developing, if it has spread, and whether it is likely to return after treatment must be taken into consideration when treating cancer. Personalised treatment planning is increasingly important for cancer diagnosis and therapy for better outcomes. A few of the revolutionary ways that this technology may lengthen life and make more cures possible include the timing of restaging and surveillance tests, the dosages of systemic-cancer medicines and radiation, and the choice and sequencing of diagnostic and treatment measures. Automated contouring of tumour targets by DL remains a significant difficulty due to the diversity of tumour shapes, locations, and interior morphologies. Even so, automated contouring expedites the procedure and enhances the uniformity of radiation oncologists [27].

With the help of AI, the development of treatment choices over the past 50 years has significantly extended the life expectancy of many cancer patients. This has been made feasible by imaging technologies that allow for early detection, highly personalised radiation therapy, targeted chemotherapies built on understanding of the human genome, and immunotherapies. Image-based ML methods can effectively predict high-risk cancer lesions. The radiotherapy workflow entails volumetric imaging, segmentation of the target volume and organs at risk (OAR), treatment planning, delivery of the treatment, and follow-up. Quality assurance (QA), when referring to the radiation workflow, is the final stage of confirming the treatments’ clinical acceptability and establishes the effectiveness of the total workflow [60]. One of the most crucial components of radiation is the contours of the treatment targets and organs at risk since the effectiveness of these contours influences both the therapeutic and unfavourable effects of the therapy [61].

According to MIT researchers, sensor technology and Internet of Things (IoT) will gather a lot of personal data that may be utilised to create individualised treatment regimens. Better diagnosis will result from it, and we will be able to use AI and ML to evaluate the vast amounts of data and choose the first and most effective course of action. The capacity to use individual biology rather than population biology at every stage of a patient’s medical journey boosts the effectiveness and speed of recovery when it comes to precision medicine. This entails gathering personal information from people, such as genetic information, physiological monitoring data, or EHR data, and customising their treatment plan in accordance with the conclusions drawn from AI models. Precision medicine has benefits, such as lower healthcare expenditures, less adverse drug reactions, and increased drug action efficacy [62]. Prescribers are assisted by more complex analytical algorithms and decision-making tools in their delivery of precision-focused and targeted therapy for a required patient.

One can avoid overtreatment and pointless surgical removal of malignancies with the use of AI. According to a study in the Journal of the National Cancer Institute, AI algorithms can identify precancerous lesions in cervical pictures and differentiate them from other abnormalities to save patients from receiving unnecessary treatment for minor problems. Glioblastoma is an acute cancer type found in the brain or spinal cord. When treating such aggressive tumors, the doctors may administer higher amount of medication and radiation to shrink the tumor, but within the safe limits for the patient. AI can help the doctors administer safe but accurate doses of medication. Here, AI could aid with the metrics and predictions, whereas IoT can help sensor-driven data generation and insight. Together, AI and IoT can optimize safe drug delivery.

Researchers from the Harvard University and the University of Pennsylvania created a DL algorithm for tumour categorization in order to treat brain cancers. Without intrusive procedures that would usually be necessary, it can identify and characterise the isocitrate dehydrogenase (IDH) mutations from simply MRI scans of gliomas. Today’s clinical practice relies heavily on guided needle biopsies and pathology updates since they can reduce the number of unnecessary surgical excisions. Robotic surgery allows for a quicker recovery and return to regular lives. Using robotic arms that enter the body through tiny incisions, procedures that traditionally required cutting a long incision from the navel to the pubic bone can now be performed on patients who need to have their prostate gland removed because they have prostate cancer. Using a specialised console, the surgeon can control the arms while seeing a magnified view of the surgical site in real time. In the prostatectomy example, a patient could leave the hospital a day after the surgery because the robotic surgery reduces blood loss and pain. While the robotic arms may seem like something out of a sci-fi movie, their delicate, precise movements might make all the difference in a situation where few millimetres could mean the difference between eliminating all malignant tissue and potentially damaging healthy tissue.

Big data and AI enable medical professionals to examine a variety of data about the patient and the cancer cells to develop individualised treatments. The side effects from this kind of therapy will be less severe. Less harm will be done to healthy cells, but it will have a greater effect on cancer cells. Together with Chicago-based AI and precision medicine company Tempus, Cedars Sinai Cancer developed molecular twins of cancer patients for use in cancer treatment. They are practically identical twins of those people. They contain data like DNA, RNA, and proteins, and aid to determine the best course of cancer treatment for a specific patient. The ability to comprehend causality rather than just correlations and weightings of medical data variables is supported by Abzu’s AI technology, which increases the possibility of making new scientific discoveries [63]. With the help of Abzu’s technology, it has been possible to pinpoint the gene combinations in breast-cancer patients that are more likely to cause mortality. These insights also make it possible to create breast-cancer treatments that are more focused and efficient. By decreasing failure rates in compound-structure activities and employing the understanding they have gained from their explainable models, Abzu’s QLattice has also been beneficial in accelerating the creation of RNA drugs. In addition, the use of AI to evaluate massive amounts of multiomics data, such as exome, transcriptome, and epigenome, along with clinically annotated datasets has produced new insights into the biology of cancer, the discovery of variants, and the prediction of RNA splice sites.

Previously, people believed that only the malignant tissue itself needed to be treated.The tissues of the lungs are treated if someone develops lung cancer. However, in the current situation, oncologists have learned that treating the cancer’s genomes rather than its tissues is more crucial to preventing recurrence, such as identifying which genes in the genome have gone awry. The vast volume of data on this topic creates numerous obstacles for proper interpretation, which AI attempts to overcome.

By offering an easy way to navigate all the research data accessible, a team of Microsoft researchers is utilising ML and NLP to assist the top oncologists in the world in determining the most effective, tailored cancer treatment for their patients [56,64]. They are working harder to understand how genetics affects cancer development and treatment. According to Fisher, “cancer may not cease to exist but once you learn how to control it, it’s a solved problem.” They contend that the best way to achieve this is through using technology to better understand cancer in general and the biological mechanisms that give rise to malignant cells. Finding the source of the issue and learning how to resolve it come next. Fisher’s objective is to comprehend the cell’s programming, which directs a cell’s actions. Once the operation of a healthy cell is understood, a diseased cell can be contrasted with it. This can assist in identifying the source of problems and their resolution. As part of BMA, a computational model that contrasts the biological functions of a healthy cell with the aberrant functions that take place when illness occurs is created. As a result, researchers may be able to observe the interactions between millions of genes and proteins in the human body that contribute to cancer and swiftly come up with the most effective and safest method of treating each patient specifically. Clinicians might submit all the biological data regarding that patient into the system using BMA. The system may then be used to conduct a variety of tests, comparing the information from the cancer patient with that of a healthy patient, for instance, or simulating how the patient’s body could react to different treatments [56]. Because there are so many variables among the millions of chemicals, proteins, and genes that interact within the human body, these calculations cannot be performed using straightforward programmes.

Repurposing drugs and innovative drug designing is another significant area where AI has contributed substantially. AI is able to forecast how various drugs would affect malignant cells. This information aids the creation of new anticancer medications and the timing of their use. Drug development is an expensive and time-consuming process, but AI can use neural networks to boost efficiency. Combining DL with reinforcement learning, which approximates the statistical link between potential actions and outcomes, results in a revolutionary approach for the discovery of innovative medicinal compounds. From regulatory processes to pharmacovigilance, AI has the potential to transform the entire pharmaceutical lifecycle. Based on various AI approaches, numerous computational tools have been suggested for cancer-related drug discovery. DeepChem, DeepTox, gene2drug, STITCH, AlphaFold, DeepNeuralNetQSAR, and so on are a few examples of the applications. For example, the Deep Tox algorithm computationally predicts 12,000 medicines and environmental contaminants for 12 different harmful effects in assays that are specially constructed [41].

According to Tao Wang from the UT Southwestern Medical Center, our immune system’s T cells are continually on the lookout for indications of cancer and other foreign invaders [53,65]. These cells bind with each other when they detect neoantigens. Neoantigens can cause cancer, yet some of them go unnoticed. The types of neoantigens number in the tens of thousands. It is difficult, expensive, and time-consuming to examine their capacity to activate T cells’ response. This is getting easier with the aid of ML.

## 5. Challenges

### 5.1. Technical Challenges

Despite numerous advantages, AI faces various challenges and constraints that prevent it from operating at full capacity in cancer research. To radically alter oncology procedures at various scales, the new wave of innovation comes with many difficulties that must be overcome. Regulation, payment, knowledge, practical difficulties, and inflexible healthcare systems are some of the obstacles in the proper adoption of AI. Labelled data are essential for training the AI classifier and predictor models. Although raw data can be simply fed into AI models, datasets still need manual annotation or, at the very least, curation. To accurately evaluate the data labels, multiple subject experts should be involved in the data-annotation process. The absence of standardised data on cancer-related health is a significant obstacle in the development of AI models, as is the lack of uniformity in the collection and storage of unstructured data inside an EHR or unified data platform of a single healthcare system.

The need for inclusive and diverse datasets for training is a significant barrier to the widespread adoption of AI algorithms and decision-support systems in the provision of cancer care. The lack of data needed to train the model is one of the most frequent problems encountered. To properly train the model better than the limited one, the majority of powerful AI models need a big sample size. High dimensionality refers to a large number of features compared to the number of medical data records included in the data. Although there are dimensionality-reduction and feature-selection methods for dealing with various dimensions to address this issue, the proper application of these approaches is essential for better outcomes. Typically, there is an unequal distribution of classes in medical data sets, particularly cancer data. A mismatch in the sample sizes of each class leads to class imbalance. Models for classifying data have a propensity to favour the class containing the majority of samples. The majority of the currently used methods successfully manage imbalance on binary classes but fall short in multi-class patterns.

Numerous studies have shown outstanding cancer-prediction results with AI. The need for a computationally efficient feature-selection approach still exists, nevertheless, in order to do away with data-cleaning operations and produce high cancer-prediction accuracy. It is necessary to refocus research on enhancing the model’s generalizability. The majority of studies have suggested a prediction model that has been tested on just one region. The models must be validated over a variety of sites in order to increase their generalizability. Reproducibility of AI-model output is difficult to achieve when applied to different healthcare systems and international communities, as was previously mentioned. However, AI algorithms and sophisticated decision-support systems are prone to data drift over time, which can have an impact on their performance, even within the setting in which they were built. According to experts, using AI and ML in treatment or diagnosis continuously might be harmful since distributional shifts may happen, meaning that target data may not match the continuing patient data used for training the model and may result in incorrect findings. Changes in technology, the healthcare system, and the population, such as gene pool, are likely to affect the relationship between the data items.

Despite being widely used in cancer research, the models’ actual application in clinics is not proactively taken into account. To help the medical expert in confirming the diagnosis decisions, these models’ predictions frequently need to be verified in a clinical context. Significant problems with data openness and interpretability brought on by AI’s “black box” process, as well as an inherent bias towards minority cohorts that limits the reproducibility of AI models and perpetuated healthcare disparities, have prevented broad implementation in clinics. Using effective and efficient optimizers, thoughtful initialization techniques, and activation functions based on local competition are some ways to solve deep architectural difficulties. However, deep network training still has a number of problems that need to be resolved because of the stacking of several non-linear transformations. Though there are approaches to identify the bias in model training, their effectiveness still needs improvement. About 90% of the research has validated DL methods over other strategies for cancer prediction using medical imagery. The DL-based techniques, however, are extremely complicated. The CNN classifier was used in about 41% of the experiments, and while it performed admirably, it did so at a considerable computational expense [65]. Furthermore, the deployment of AI in many developing nations can be hampered by the absence of knowledge in the computing algorithms and the technologies by therapists and doctors.

Today’s clinical-diagnostic AI algorithms are referred to be weak AI. In other words, they lack broad intelligence, are not adaptable enough to handle additional clinical diagnostic tasks, and are trained to do a specific activity. However, a fully trained AI algorithm can be modified to carry out closely related tasks using transfer-learning techniques.

### 5.2. Ethical Challenges

Since right and wrong are subjective, ethical values are also subjective. An AI system’s accuracy and consistency are often limited by its training data and the hardware used. We should always remember that AI can and will make faulty decisions in some situations because its decision making is predictive and probabilistic in nature. As such, there are no regulations or guidelines to establish who is legally accountable when AI malfunctions or causes harm in the course of providing a service. Another aspect is that high-income and resource-driven centres have been the majority of places where the actualization of healthcare AI’s potential has been evaluated. Oncological, AI-based prediction tools are projected to have a greater impact and increase efficiency when used in low-resource and rural areas with a shortage of skilled physicians and specialists. It can be difficult to ensure that the AI tool for cancer detection and treatment does not have any rooted bias because many clinics buy the software model from corporations. To make the AI models work effectively and accurately, an enormous amount of data will be needed. People whose data are used for model development become anxious due to improper transparency in data usage. Data security and privacy are now more at stake. The use of therapeutic chatbots, avatars, and social-assistive technology raises ethical questions primarily about long-term uses that may result in patient dependence.

Additionally, the incorporation of AI technologies into daily life and healthcare is altering moral standards and social expectations. Payors are becoming increasingly concerned about the rising expense of treatments resulting from curative-therapy combinations, particularly when those treatments might only be effective for a portion of the population, as is the case with checkpoint inhibitors. Value-based strategies are being developed and will have an impact on the uptake and payment of these therapies. The delivery of a specific message will be the main technological impediment for emerging therapeutic techniques. Operational consistency is the main hurdle to achieve adequate robustness and sensitivity for diagnostics across a variety of geographical locations [66]. Protocol standardisation and training are necessary to guarantee the necessary skill sets needed. Patient enrolment for clinical research will become more difficult. Because of the numerous studies that run concurrently, it is challenging to recruit individuals for indications of high incidence because there is such intense competition for them. Smaller, niche populations are difficult to locate as well because it might need a network of hospitals to bring in enough patients.

Clinical practice at academic medical centres and community hospitals still differs significantly today. These differences are dwindling in the United States, in part due to the integration of oncology practices by integrated health networks and in part due to new businesses (like Flatiron Health, Foundation Medicine, IBM Watson, NantHealth, and Syapse) that streamline oncology work flows, integrate genomic data and EHRs, and translate these data into understandable reports that can be used to take appropriate actions [19,64,66]. The speed at which change can be implemented will be capped by any systems-level fragmentation.

## 6. Future Directions

The scope of AI to cancer will go beyond prevention, diagnosis, and therapy to include daily life [10]. Every proposed modern technology goes through a hype cycle. The Technology Trigger phase, the Peak of Inflated Expectations phase, the Trough of Disillusionment phase, the Slope of Enlightenment phase, and the Plateau of Productivity phase are the five phases of a hype cycle. An impediment to the actual realisation of a technology’s utility results from a technology’s development through these stages. With increased effort and cooperation from specialists in research, medical, government, and community implementation, the aforementioned hurdles can be overcome. Focus must be shifted away from cancers with high incidence like lung, colon, and breast, and toward malignancies with lower medical progress. The entire wheel is undergoing innovation that will influence cancer care in the future.

Additionally, these platforms may be used in conjunction with other digital technologies, such as telemedicine for virtual consultations and the Internet of Medical Things (IoMT) to enhance referral practises. AI can be used to determine the precise risk stratification of cancer stage and decide the appropriate course of treatment. When analysing data, common determinants exist at many different levels that can be used by AI models to identify the causative connections between variables. Integrated knowledge can improve the accuracy of AI research on specific cancer-related medical occurrences while also enabling the interpretability of AI models. An emerging trend is explainable AI and interpretable DL [41]. Explainable AI explains a model’s functioning, strengths and weaknesses, likely behaviour, and potential biases to a specific audience while enabling accuracy, fairness, accountability, stability, and transparency in decision making. Interpretability is a concern for DL models as these models achieve high accuracy at the expense of high abstraction. The supporters and opponents of AI in healthcare have historically divided the field. The fundamental requirement for success is that both biologists and computer scientists must apply their specialisation knowledge for the success of these models. Cancer Moonshot is a cancer community that has gathered a sizable network of patients, advocates, researchers, and physicians who are committed to furthering research to enhance the lives of those with cancer and those who are at risk [67]. Their objectives include promoting more collaboration, improving the exchange of cancer data, and accelerating scientific discoveries in the field of cancer.

Customization is the key for cancer treatment in the future. In the far future, there will be living databases that compile every facet of a person’s health into discrete data elements. These databases will power extremely complex models that can customise therapy selection, dose calculation, surveillance modality and schedule, and other things. For more individualised cancer-treatment strategies or precision medicine, BMA holds enormous promise. Researchers are hoping that a system like BMA could eventually allow researchers and oncologists to thoroughly examine a person’s cancer case and also run tests that take other factors into account that could affect treatment, such as comorbidity of the patient or whether the patient is taking non-cancer medications that might interact with the cancer drugs. BMA use tools that aesthetically resemble those that researchers may use in the lab and speaks in a language that biologists can comprehend [56]. Self-diagnostic mobile applications, for instance, SkinVision, were created to help users check for cancer-in-skin anomalies. They can use the camera on their smartphone to take a picture of a suspicious spot on their skin and send it for analysis. Within 30 s, an AI programme will evaluate the lesion’s colour, texture, and shape, and provides feedback to the user. This system is providing about 95% accuracy in detecting skin cancer [53].

Biologist Neil Dalchau wants to explore the possibility of coding with cells [56]. He is part of a team that is trying to learn and do computing in cells instead of silicon. Their team intends to use biological systems to compute cell activities while applying what they have learnt about conventional computing to biotechnology or medicinal applications. Building a molecular computer that can be placed inside a cell to check for illness has been offered as one solution to this problem. The sensor would activate an anti-disease response if it discovered a disorder. This is a significant advancement over many current cancer treatments, which result in the destruction of good cells while attempting to combat cancerous ones. But in this instance, unlike the cell and many of its intricate internal workings, the specially constructed molecular computer is regulated to function as intended. Programming a cell effectively requires a thorough understanding of how it computes. A futuristic concept is to equip immune networks to deal with flaws or bugs in healthy cells that cause cancer. In this type of collaboration, biologists with expertise in the field might offer theories that can guide computer scientists in what to look for in the data. In turn, computer scientists can perform the analyses required to support them in supporting or refuting their hypotheses.

Digital pathology, customised biopsies, and imaging are all examples of diagnostic technologies. The breadth of therapeutic technologies includes conventional methods based on small and big chemicals, their derivatives, radiation, as well as nucleic acid-based treatments, such as vaccinations and cell therapies. The integration of AI with the new therapeutic modalities, including improved antibody constructions, cell treatments, nucleic-acid therapies, and cancer vaccines, will open up new therapy options in the future. The use of combination immunotherapies continues to garner media attention. This is shown by the rise of combination therapy in oncology, with 81% of immuno-oncology (IO) trials using combinations of two or more drugs [66].

Previously difficult to treat B-cell malignancies like relapsed- and refractory-acute- lymphoblastic leukaemia are now being treated with outstanding success with cell treatments like CAR-T. The “activation” and “kill” switches are included into the second and third generations of CAR-T therapies to further enhance specificity, safety, and amplification in collaboration with AI. However, recent failures brought on by unfavourable circumstances have generated fresh scepticism about the future of important programmes. In general, whether cell treatments can be as successful against solid tumours is still up for debate [7,8,67].

Clustered regularly interspaced short palindromic repeats (CRISPRs) is a high-precision, gene-editing technique that has gained popularity in preclinical research and has the potential to offer a permanent cure. The most cutting-edge clinical uses for CRISPR centre on how it can be used to support cell treatments. To reduce the immunogenic potential of allogeneic transplantation, CRISPR may potentially be employed to edit T-cell receptors or major histocompatibility complexes out of the genome. The ability to swiftly and simply alter the genetic code of living cells was never anticipated by researchers. CRISPR functions like a pair of scissors and can accurately remove, insert, or modify particular sections of DNA inside cells. This ground-breaking gene-editing technique was discovered as a result of a side study motivated by an interest in how bacteria defend themselves against viruses.

More research is being done on CRISPR-made cancer medicines, and recently the first US-clinical study of a CRISPR-made cancer immunotherapy was underway. Trials to use CRISPR directly in the body are also beginning. CRISPR is a game-changer, but technology still has drawbacks, and there is still ongoing discussion over the morality of gene editing. The fact that the CRISPR-editing tool often induces mutations into double-stranded DNA during the repair process is a significant issue. The targeted sequence and the guide RNA (gRNA), among other variables, are thought to be important influences on the errors, but they also appear to have a repeatable pattern. Researchers at the Wellcome Sanger Institute claim to have created a technique that uses ML to foretell cell changes that CRISPR will cause. This could enhance the efficacy of CRISPR research and makes it simpler to translate it into viable and safe therapies [68]. Researchers from the School of Medicine at the University of Washington in Seattle stated that it is feasible to activate specific dormant genes by turning off the chemical “off switches” that keep them silent by merging CRISPR technology with a protein created with AI. This strategy will enable researchers to comprehend the function-specific genes that serve in both healthy cell growth and development, and malignancy [69].

New tools against targets that are challenging to treat with small compounds and biologics are offered by RNA therapies. Transiently suppressing transcription, creating internal or cell-surface proteins, and adding missing or down-regulated proteins are a few examples of these. Onkaido Therapeutics and Silence Therapeutics from Moderna Therapeutics are two businesses that have already engaged in the development of cancer medicines. The initial products are probably going to concentrate on a few simple organs, including the liver and blood, or on conditions that allow intratumoral injection [66].

Customizing medicines based on a deeper understanding of biology using AI analysis is the next stage in the personalization of cancer treatment. Neoantigen and microbiome methods have yet to reach their full potential, but they may provide new chances for indications in cases when driver mutations are unavailable or cannot yet be “drugged” by existing modalities. According to Data Sciences at UT Southwestern Medical Center, it did not appear conceivable to determine which neoantigens to attach to T cell receptors [66]. However, it is improving with ML.

A more detailed approach to monitoring disease state and therapy response is made possible by easier AI-enabled sequencing and other biomarker technologies. This will improve information accessibility for doctors, creating a critical mass and facilitating adoption, along with enhanced interaction with EHRs and real-time decision support. In order to implement precision-oncology programmes at scale across community hospitals, Syapse created a digital platform that combines genetic and clinical data analysis. Other major groups that work to increase access to precision medicine are Flatiron and IBM Watson [65,70,71].

Additional cancer development monitoring will be possible with liquid biopsy, which also offers the advantages of being less expensive and risky than traditional biopsies and has less sample problems. With its less intrusive nature, improved evaluation of response over time, and tracking of minimal residual illness, it may enhance usage in genomic evaluation of less accessible malignancies. From liquid biopsies to connected health tools like wearables and point-of-care diagnostics, a wider range of information sources will be accessible. Despite all these benefits and the obvious advancement in current practices, widely used rule-based systems show a number of drawbacks, including expensive development costs, system limitations caused by the challenging encoding of complex relationships, and a need for in-depth medical knowledge. As a result, the identification of such data patterns has improved thanks to its integration with AI systems [14].

## 7. Conclusions

In this paper, we presented the opportunities, trends, challenges, and future directions of AI in cancer research. Cancer is identified as one of the leading causes of death by WHO. Initiatives like Cancer Moonshot intends to reduce the cancer death rate by half in the next 25 years. Conventional practices for cancer diagnosis and treatment include radiology, chemotherapy, surgery, and radiation. AI models could improve the accuracy and effectiveness of the methods used for cancer prediction, diagnosis, and treatment. To develop these models, we need large datasets for training and validation, and many such datasets are available publicly. The healthcare providers can access AI services through cloud-AI platforms conveniently. AI-based classifiers are helping pathologists in diagnosing cancer accurately and consistently. Predictive-AI models are establishing a person’s likelihood of getting a certain type of cancer by identifying the risk factors. Personalised cancer treatment with the help of AI is one of the key methods for better cure outcomes. AI makes it possible for the patient to have a longer life, less pain, and more cures by appropriately controlling the timing of restaging and surveillance tests, the dosages of systemic-cancer medicines and radiation, and the choice and sequencing of diagnostic and treatment measures.

Proper adoption of AI for cancer research is constrained by regulatory controls with respect to data security and privacy, non-availability of labelled data, data bias, imbalanced data, and so on. It is a fact that AI can and will make faulty decisions in certain situations because its decision making is predictive and probabilistic. So, if AI makes a faulty decision, we cannot hold it responsible. Explainable-AI and interpretable-DL are steps towards mitigating this risk and providing rationale for decision making. Unless a human-medical expert works with the AI system, we cannot have a human-verification element in the process. Therefore, no one anticipates that AI will entirely replace medical professionals in the near future. AI-based precision medicine is going to be the key for cancer treatment in the future. Living databases will power extremely complex models that can customise therapy selection, dose calculation, surveillance modality and schedule, and so on. However, the migration from artificial narrow intelligence (ANI) to artificial general intelligence (AGI) will witness the automation of almost all processes for cancer prediction, diagnosis, and treatment.

## Figures and Tables

**Table 1:** Worldwide cancer-related deaths in 2020 (WHO, 2022).

Cancer Type	Deaths
Lung	10,000,000
Colon and Rectum	916,000
Liver	830,000
Stomach	769,000
Breast	685,000

**Table 2:** Worldwide new cancer cases in 2020 (WHO, 2022).

Cancer Type	New Cases
Breast	2,260,000
Lung	2,210,000
Colon and Rectum	1,930,000
Prostrate	1,410,000
skin (non-melanoma)	1,210,000
Stomach	1,090,000
